# Factors associated with different numbers of health behaviors by living arrangements

**DOI:** 10.1186/s12889-020-09242-y

**Published:** 2020-07-20

**Authors:** Namhee Kim, Heejung Kim, Sooyoung Kwon

**Affiliations:** 1 College of Nursing, Yonsei University, 50-1 Yonsei-ro, Seodaemun-gu, Seoul, Republic of Korea 03722; 2 Mo-Im Kim Nursing Research Institute, Yonsei University, 50-1 Yonsei-ro, Seodaemun-gu, Seoul, Republic of Korea 03722

**Keywords:** Health behavior, Living arrangements, Living alone

## Abstract

**Background:**

As the number of individuals living alone increases, it becomes clear that health disparities vary according to a person’s living arrangement. However, very few studies have investigated the characteristics of individuals who improve or maintain multiple healthy behaviors based on their living arrangements. This study aimed to explore the differing individual characteristics and multiple health behaviors in Korean adults living alone compared to those living with others and to identify the factors significantly associated with these behaviors.

**Methods:**

This study utilized a secondary analysis, using 2013–2015 Korea National Health and Nutrition Examination Survey data, with a cross-sectional and descriptive correlational design (*N* = 15,934). Multiple health behaviors, based on the comparison of past and present behaviors, included smoking, alcohol consumption, and weight control. The total number of health behaviors was calculated as the sum of each single health behavior. The different numbers of health behaviors were categorized into four levels: from 0, none of the three health behaviors to 3, all three health behaviors. Descriptive statistics and generalized ordinal logistic regression analysis were used.

**Results:**

People living alone engaged in fewer healthy behaviors (*p* <  0.05) and reported lower rates of maintenance of abstinence from smoking and weight control compared to those living with others, but they maintained a status of abstaining from alcohol consumption more than those living with others (*p* ≤ 0.001). In particular, higher self-rated health statuses (Adjusted Odds Ratio [aOR] = 2.03, 95% Confidence Interval [CI] = 1.04–3.97), being overweight (aOR = 1.46, 95% CI = 1.11–1.92), and having shorter sleep durations per day (aOR = 0.74, 95% CI = 0.55–0.99) were significantly associated with 0, 1 versus 2, 3 levels of healthy behaviors in those living alone.

**Conclusions:**

Korean adults who lived alone had different factors associated with different combinations of multiple healthy behaviors compared to those living with others. Therefore, we need to manage healthy behaviors by considering associated factors for those living alone. Specifically, clinicians should consider the vulnerability of health behaviors in people living alone and provide customized approaches and multidimensional interventions based on their living arrangements.

## Background

The World Health Organization (WHO) emphasizes the benefits of health promotion and encourages all countries and stakeholders to make active efforts to encourage health behaviors [1]. Health behaviors are considered to be patterns, actions, or habits of behaviors that are associated with maintaining one’s health status, restoring health conditions, and changing one’s health status via life modifications using self-motivation [2]. Healthy behaviors play an important role in a person’s health promotion [3] and in reducing avoidable disease burdens, such as non-communicable diseases and their potential morbidity, mortality, and disability [4]. The Centers for Disease Control and Prevention (CDC) focused on enhancing modifiable behaviors, including cigarette smoking, alcohol consumption, and weight control, which were associated with mortality rates within the United States to reduce overall health disparities [3, 5]. In keeping with international standards [3–5], the Korean government developed healthcare guidelines (Health Plan 2020) that aimed to improve modifiable health behaviors regarding smoking, alcohol consumption, and weight control [6]. To accomplish this goal, it is necessary to understand the nature of more vulnerable groups in terms of promotion to improve healthy behaviors using a tailored approach.

In Korea, individuals living alone are a vulnerable group for experiencing various health problems, low levels of wellbeing, and overall insufficient healthy behaviors [7–10]. Compared with those living with others, adults living alone reported higher levels of chronic diseases, self-reported depression, suicidal thoughts or plans, all associated with higher usages of hospitals and outpatient healthcare services [9]. Moreover, those living alone reported higher rates of cigarette smoking and alcohol consumption [7, 9]. Previous studies investigating living arrangements as an explanatory variable [11], either only targeted individuals living alone without subgroup comparisons [12], or focused on old-age groups based on living arrangement [13, 14]. However, a person’s living arrangement is uniquely associated with different aspects of health behaviors [7, 9, 11]. Thus, it is necessary to identify the differences due to peoples’ living arrangements and develop new strategies considering these in order to effectively reduce health disparities and promote healthier behaviors.

Multiple unhealthy behaviors tend to co-occur [15–17]; thus, they need to be assessed simultaneously [15, 17]. Specifically, it is important to maintain a higher quantity of healthy behaviors at the same time and for a longer period [18, 19] rather than engaging in a single attempt of action in a temporary manner. Ramo and colleagues [20] noted that smokers showed more engagement with multiple health risk behaviors, specifically young smokers [20], and cigarette smoking, excessive alcohol consumption, and lack of physical activity tended to co-occur as a high risky behavior cluster [21]. However, previous studies have examined peoples’ past or current status of each health behavior [7, 9] or focused on single health behavior changes [11, 22]. It is necessary to understand the nature of multiple healthy behaviors that have changed over time, or those related to maintaining healthy behaviors over long periods of time [18, 19], to develop a more comprehensive health promotion program [15, 23]. However, little is known about how different factors, specifically living arrangements, are associated with multiple health behaviors. Therefore, we focused on multiple health behaviors and, in addition, the maintenance of healthy behaviors or attempts to change behaviors according to healthcare experts’ recommendations, by comparing a person’s past and present statuses. The aims of this study were to explore the different characteristics and multiple health behaviors by comparing smoking, alcohol consumption, and weight control behaviors of people living alone and those living with others and to identify factors associated with multiple health behaviors in Korean adults by comparing the two types of living arrangements.

## Methods

### Description of the primary data

This study utilized a cross-sectional and descriptive correlational design with a secondary data analysis of the sixth wave of the Korea National Health and Nutrition Examination Survey (KNHANES; 2013–2015). The primary data were collected from 2013 to 2015 and were released in 2017 by the Korea Centers for Disease Control and Prevention (KCDC). A stratified, multi-stage, clustered probability sampling design was used based on the size of the geographical area, sex, and age, based on collected household registries in order to accurately represent the Korean population. The final sample was selected from 576 regions and 11,520 households. Therefore, KNHANES contains nationally representative data, having both external validity and reliability [24]. The KNHANES consists of a health interview, health examination, and nutrition survey.

### Samples of the secondary data analysis

**Fig. 1:**
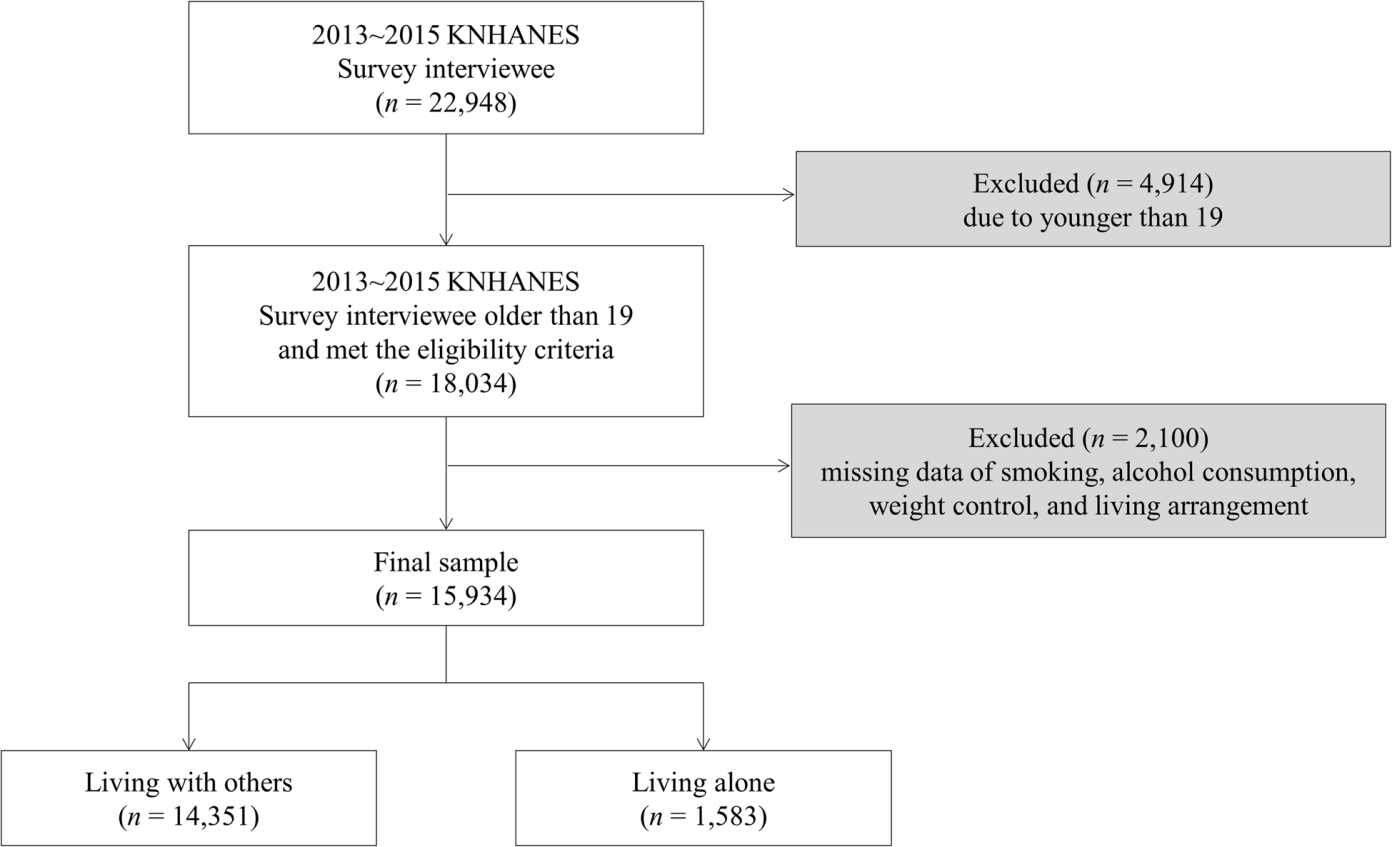
Flowchart of study samples

In total, 22,948 participants completed the sixth wave of the KNHANES. Korean Statistics Information Service reported only 1% of adolescents lived alone [25], thus, we focused on the adult group by living arrangement in our secondary data analysis. Therefore, the final sample was 15,934 after excluding those younger than 19 years (*n* = 4914), those with missing data related to smoking, alcohol consumption, and weight control behaviors (*n* = 2099), or those who did not report their living arrangement status (*n* = 1) (Fig. 1). The final sample size obtained was sufficient to perform a generalized ordinal logistic regression model based on the result of power analysis using G-power version 3.1. We calculated this sample size by using a normal X distribution, small effect size (d = 0.20), two-tailed alpha at 0.05, a power of 0.80, and odds ratio (OR) of 1.12 [21].

### Measures

We utilized a health interview and examination data from the KNHANES [24].

#### Dependent variable

Multiple health behaviors including smoking, alcohol consumption, and weight control were the dependent variables for this study. We chose these variables assuming that the maintenance of an action constitutes a valuable construct [18]. We defined the dependent variable, not just on defining healthy behaviors as “yes” or “no,” but in terms of the maintenance of healthy behaviors or attempts to change one’s behaviors as recommended by comparing a person’s past and present statuses of smoking, alcohol consumption, and weight control. Based on completed self-report, smoking was measured using two questions: “How many cigarettes have you ever smoked during entire your life?” and “Do you currently smoke?” Responses were categorized as no smoking (coded as 1; *Never smokers* or *ex-smokers who either smoked less than 100 cigarettes in their lifetime or smoked 100 cigarettes or more in their lifetime but who are currently non-smokers*) or smoking (coded as 0; *current smoker*). Alcohol consumption status was measured using two questions: “Have you ever consumed any alcohol during your entire life?” and “How often did you consume alcohol during the past year?” Responses were dichotomized as no alcohol consumption (coded as 1; *no drinking alcohol during one’s lifetime, no drinking alcohol during the past year,* or *drinking alcohol less than once per month during the past year*) and alcohol consumption (coded as 0; d*rinking alcohol once or more often per month during the past year*). Weight control was assessed according to responses to the following question: “Have you ever tried to control your weight on your own during the past year?” Responses were categorized as weight control (coded as 1; *tried to lose weight, tried to maintain current weight,* or *tried to gain weight*) or no weight control (coded as 0; *never tried to control weight*). We summed the total number of health behaviors, resulting in a range from 0 to 3 (0 = none of the three selected health behaviors, 1 = any one of the three selected health behaviors, 2 = any two of the three selected health behaviors, and 3 = all three selected health behaviors).

#### Independent variables

We included socio-demographic and health-related characteristics as the independent variables, which were selected and categorized based on a literature review for a comparison of this study’s results against those of previous studies [24, 26–31]. Socio-demographic characteristics included participants’ age, sex, marital status, education and economic level, employment status, and living arrangement. Age was categorized into three groups: 19–39 years, 40–64 years, and over 65 years [28]. Sex was dichotomized as male or female. Marital status was categorized as single or married. Participants’ education level was categorized into one of three groups: elementary school or lower, middle to high school, and college or higher, taking into consideration the normative levels of the Korean educational system [24, 31]. Economic level was classified into three groups: lowest, lower-middle, or upper-middle, and highest [30] by dividing the quartiles of total household income levels by the total number of family members in each [24]. Employment status was categorized as unemployed/non-economic activity or employed [24]. Living arrangement was asked using the question: “Which of the following is your household type?” Responses were dichotomized as those living alone (one-person household) and those living with others (two-or-more-person households) [24].

Health-related characteristics included participants’ self-rated health status, body mass index (BMI), diagnosis of chronic diseases and depression, stress, and sleep duration per day. Self-rated health statuses were measured via on the question “How would you rate your current health status?” and was categorized into five groups: very poor, poor, fair, good, or very good [24]. BMI was categorized into three groups using the Asia-Pacific obesity diagnostic criteria: underweight (BMI < 18.5 kg/m^2^), normal (18.5 ≤ BMI < 25 kg/m^2^), and overweight (BMI ≥ 25 kg/m^2^) [27]. Medical diagnoses of chronic diseases and depression were self-reported, with chronic diseases including hypertension, hyperlipidemia, stroke, heart disease (myocardial infarction or angina), osteoarthritis, diabetes mellitus, thyroid disease, renal failure, hepatitis B, hepatitis C, hepatocirrhosis, tuberculosis, asthma, atopy, rhinitis, or cancer (gastric, liver, colon, breast, cervix, lung, thyroid, or other) [24, 29]. Stress levels were measured via the question “How much do you feel stressed in your daily life?” and were then categorized into two groups: low (feel hardly any stress at all or only a little bit) and high (feel stressed daily quite or very much) [24]. The sleep duration per day was self-reported, measured in the form of average hours per day, and categorized into three groups: 0–5 h, 6–8 h, and 9 or more hours [26].

### Statistical analysis

Statistical analyses were performed using the IBM SPSS version 24 and Stata 13. To gain an accurate representation for the non-institutionalized civilian Korean population without biased estimates, we assigned the integrated weighting of a 3-year period (2013–2015) and generated complex sample designs using IBM SPSS Complex Samples. In all analyses, we considered a two tailed statistical level of significance of *p* = 0.05. We did not conduct an imputation [32], as most of the missing data were composed of either “unknown” or “systematic missing” [24], and came to less than 5% of the total.

Chi-squared tests were performed using complex sample analysis in order to compare the differences between the data for socio-demographic and health-related characteristics related to multiple health behaviors depending on living arrangements and to compare the differences in the participants’ characteristics according to single healthy behavior. Generalized ordinal logistic regression was conducted using the Stata package *gologit2* command in order to estimate the factors associated with multiple health behaviors by varying living arrangements. An unweighted sample was used for the generalized ordinal logistic regression after controlling for the socio-demographic characteristics (age, sex, marital status, education and economic level, and employment status).

#### Generalized ordinal logistic regression

The dependent variable in this study was categorized into four levels: from 0 = none of the three selected health behaviors to 3 = all three selected health behaviors. These four category levels of the dependent variable are ordinal, thus the ordered logit model is recommended [33, 34]. However, the ordered logit model assumes proportional odds and parallel lines. When these assumptions are not feasible, a generalized ordinal logistic regression is often used as an alternative. The generalized ordinal logistic regression model can estimate a partial proportional odds version. In addition, it gives information about each comparison made via providing a distinctive set of regression coefficients, which means that the effect of each independent variable is not equal, according to the change of the ordinal dependent variable. Therefore, the advantage is that the threshold-specific effects of the independent variables violating the proportionality assumption can be estimated. In this study, the generalized ordinal logistic regression model had three coefficient sets (0 level versus 1, 2, and 3 levels; 0 and 1 levels versus 2 and 3 levels; 0, 1, and 2 levels versus 3 level) which enabled a comparison of all category levels against all of the others. Therefore, a generalized ordinal logistic regression analysis is able to identify all differences due the impact of each independent variable on the dependent variable [33, 34]. By examining the direction and magnitude of the effects of the associated factors on the level of health behaviors, it deepens the understanding of this theoretical phenomenon, links the results to effective nursing interventions, and predicts more accurate experimental effects.

### Ethics approval

The KNHANES was approved by the Institutional Review Board (IRB) of the KCDC (2013-07CON-03-4C and 2013-12EXP-03-5C). All study participants within the primary study provided informed consent. The KNHANES data is publicly available, and is available for all university researchers, for scientific purposes, at a no charge. This study was approved by the IRB of Yonsei University (Y-2017-0020).

## Results

### Characteristics of participants according to living arrangement

Among the participants, 92.0% of them lived with others whereas 8.0% lived alone. People living alone showed different characteristics when compared against those living with others. For the socio-demographic characteristics, people living alone demonstrated higher proportions of advanced age, being female, single marital status, poor education or economic status, and unemployment when compared to those living with others (all *p* values < 0.05). For the health-related characteristics, people living alone reported higher rates of poor self-rated health statuses, diagnoses of chronic diseases, depression diagnoses, and shorter sleep durations per day than those living with others (all *p* values < 0.001; see Table 1).

**Table 1:** Characteristics of participants by living arrangement

Variables	Categories	Total	Living with others	Living alone	*P-*value
Total		100.0%^a^ (*N*^b^ = 15,934)	92.0%^a^ (*n*^b^ = 14,351)	8.0%^a^ (*n*^b^ = 1583)	
**Socio-demographic characteristics**					
Age	19–39	38.2	39.1	28.4	< 0.001
	40–64	46.7	47.8	33.8	
	65 and older	15.1	13.1	37.8	
Sex	Male	49.1	49.4	45.0	< 0.05
	Female	50.9	50.6	55.0	
Marital status	Single	22.7	21.5	36.5	< 0.001
	Married	77.3	78.5	63.5	
Education level	Elementary school or lower	16.6	14.6	38.8	< 0.001
	Middle to high school	47.3	48.3	36.1	
	College or higher	36.1	37.1	25.1	
Economic level	Lowest	15.3	12.8	44.7	< 0.001
	Lower-middle, or upper-middle	54.2	55.5	39.4	
	Highest	30.5	31.7	15.9	
Employment status	Unemployed/non-economic activity	37.4	36.6	46.7	< 0.001
	Employed	62.6	63.4	53.3	
**Health-related characteristics**					
Self-rated health status	Very poor	3.3	2.9	8.9	< 0.001
	Poor	13.8	13.2	20.2	
	Fair	50.8	51.3	45.1	
	Good	27.0	27.5	20.7	
	Very good	5.1	5.1	5.1	
BMI	Underweight	4.8	4.8	4.0	0.130
	Normal	62.8	63.0	61.0	
	Overweight	32.4	32.2	35.0	
Diagnosis of chronic diseases	Yes	49.1	47.8	63.6	< 0.001
	No	50.9	52.2	36.4	
Diagnosis of depression	Yes	4.3	3.9	8.9	< 0.001
	No	95.7	96.1	91.1	
Stress	Low	73.7	73.8	72.4	0.300
	High	26.3	26.2	27.6	
Sleep duration per day	≤ 5 h	15.1	14.2	25.7	< 0.001
	6–8 h	77.9	78.8	67.4	
	≥ 9 h	7.0	7.0	6.9	

Note: The missing data was less than 5% and non-responses were excluded from the analysis. *P*-values were determined using Chi-square tests with complex sample analysis

*BMI* Body mass index

^a^Weighted percentages calculated by a complex sample analysis

^b^Unweighted number

### Comparison of multiple healthy behaviors according to living arrangement

Additional participants’ characteristics related to a single healthy behavior are reported in Additional file 1. Different combination of multiple healthy behaviors were compared in Table 2. Maintenance of smoking abstinence and weight control had higher rates in those living with others than those living alone (both *p* values ≤ 0.001), whereas the rates of alcohol abstinence were higher in those living alone (*p* <  0.001). In terms of the sum of multiple healthy behaviors, a total of at least two types of selected health behaviors was the highest proportion regardless of living arrangement (43.9% in those living with others and 45.5% in those living alone). However, those living with others performed more selected healthy behaviors than those living alone. The rates of none of the healthy behaviors were slightly higher in those living alone (9.4%) than those living with others (7.3%), whereas the rate of all three multiple health behaviors was 24.7% in those living with others, and 22.1% in those living alone, respectively (*p* values < 0.05; see Table 2).

**Table 2:** Comparison of multiple healthy behaviors by living arrangement

Variables	Categories	Total	Living with others	Living alone	*P-*value
Total		100.0%^a^ (*N*^b^ = 15,934)	92.0%^a^ (*n*^b^ = 14,351)	8.0%^a^ (*n*^b^ = 1583)	
**Maintenance of health behaviors**					
No smoking	Yes	76.9	77.3	72.0	0.001
	No	23.1	22.7	28.0	
No alcohol consumption	Yes	41.6	41.0	48.9	< 0.001
	No	58.4	59.0	51.1	
Weight control	Yes	67.0	67.6	59.3	< 0.001
	No	33.0	32.4	40.7	
**Numbers of healthy behaviors**					
0		7.5	7.3	9.4	< 0.05
1		24.0	24.1	23.0	
2		44.1	43.9	45.5	
3		24.4	24.7	22.1	

Note: The missing data was less than 5% and non-responses were excluded from the analysis. *P*-values were determined using Chi-square tests with complex sample analysis

^a^Weighted percentages calculated by a complex sample analysis

^b^Unweighted number

### Factors associated with multiple healthy behaviors according to living arrangement

Table 3 shows the result of the generalized ordinal logistic regression used to determine the factors associated with individual’s maintenance of multiple healthy behaviors according to living arrangement. The associated factors showed different directionalities and magnitudes in their coefficients for each group. A “very good” self-rated health status (Adjusted OR [aOR] = 2.03, 95% confidence interval [CI] = 1.04–3.97) was the most significant factor, with the only sizeable effect, in people living alone. Being “overweight” was associated with increased numbers of healthy behaviors for both living arrangement groups in each comparison of the category levels. In particular, the aORs for the results of all category levels in people living alone showed more significant and positive effects than that of those living with others. In the case of the comparison between the 0 level versus 1, 2, and 3 levels, the magnitude of the aOR of being overweight in people living alone (aOR = 2.39, 95% CI = 1.42–4.01) had a much higher positive effect than that of those living with others (aOR = 1.59, 95% CI = 1.35–1.87). Short sleep durations per day (≤ 5 h) (aOR = 0.74, 95% CI = 0.55–0.99) was associated with moderate maintenance of multiple healthy behaviors (0 and 1 levels versus 2 and 3 levels) in those living alone. However, both short (aOR = 0.87, 95% CI = 0.77–0.98) and long sleep durations per day (aOR = 0.82, 95% CI = 0.70–0.96) categories were associated with moderate maintenance of multiple healthy behaviors (0 and 1 levels versus 2 and 3 levels) for those living with others (see Table 3).

**Table 3:** Generalized ordinal logistic regression result of multiple healthy behaviors by living arrangement

Variables	Living with others (*n* = 13,599)			Living alone (*n* = 1485)		
	Numbers of healthy behaviors			Numbers of healthy behaviors		
	0 vs 1,2,3	0,1 vs 2,3	0,1,2 vs 3	0 vs 1,2,3	0,1 vs 2,3	0,1,2 vs 3
**Health-related characteristics**						
Self-rated health status (ref. Fair)						
Very poor	0.99 (0.62–1.59)	1.42 (1.10–1.84)^**^	1.21 (0.98–1.50)	1.15 (0.43–3.10)	1.34 (0.79–2.28)	1.20 (0.80–1.82)
Poor	0.77 (0.62–0.96)^*^	0.93 (0.82–1.05)	1.13 (1.01–1.27)^*^	1.40 (0.77–2.55)	0.87 (0.62–1.21)	0.79 (0.57–1.10)
Good	1.37 (1.14–1.64)^**^	1.24 (1.12–1.37)^***^	1.04 (0.95–1.15)	1.67 (0.92–3.00)	1.16 (0.83–1.63)	0.99 (0.70–1.39)
Very good	1.20 (0.85–1.68)	1.08 (0.90–1.31)	1.08 (0.88–1.31)	1.69 (0.61–4.70)	2.03 (1.04–3.97)^*^	1.26 (0.67–2.36)
BMI (ref. Normal)						
Underweight	0.82 (0.54–1.24)	0.70 (0.56–0.83)^***^	0.85 (0.70–1.04)	7.09 (0.93–53.80)	1.10 (0.55–2.22)	0.68 (0.30–1.52)
Overweight	1.59 (1.35–1.87)^***^	1.41 (1.29–1.54)^***^	1.37 (1.26–1.49)^***^	2.39 (1.42–4.01)^**^	1.46 (1.11–1.92)^**^	1.38 (1.07–1.77)^*^
Diagnosis of chronic diseases^a^ (ref. No)						
Yes	1.38 (1.18–1.61)^***^	1.25 (1.14–1.36)^***^	1.14 (1.04–1.24)^**^	1.47 (0.92–2.36)	1.22 (0.91–1.64)	1.27 (0.92–1.74)
Diagnosis of depression^a^ (ref. No)						
Yes	1.05 (0.63–1.73)	0.94 (0.76–1.18)	1.13 (0.95–1.36)	1.03 (0.42–2.56)	1.55 (0.93–2.57)	1.36 (0.90–2.07)
Stress^a^ (ref. Low)						
High	0.81 (0.69–0.96)^*^	0.76 (0.69–0.83)^***^	0.82 (0.74–0.90)^***^	0.93 (0.56–1.55)	1.00 (0.74–1.34)	0.84 (0.63–1.13)
Sleep duration per day (ref. 6–8 h)						
≤ 5 h	1.06 (0.85–1.32)	0.87 (0.77–0.98)^*^	1.01 (0.90–1.12)	0.70 (0.40–1.20)	0.74 (0.55–0.99)^*^	0.89 (0.67–1.18)
≥ 9 h	0.77 (0.58–1.02)	0.82 (0.70–0.96)^*^	0.87 (0.74–1.02)	0.96 (0.33–2.84)	1.06 (0.63–1.79)	0.84 (0.52–1.34)

Note: An unweighted sample was used for the generalized ordinal logistic regression. The missing data was less than 5% and non-responses were excluded from the analysis. Values are adjusted odds ratio (95% confidence interval). Numbers in bold indicate significant value

*BMI* Body mass index

^a^Dichotomized variables

^*^p < .05, ^**^p < .01, ^***^p < .001

## Discussion

This secondary data analysis, using the 2013–2015 KNHANES data, identified differences in both health-related characteristics and multiple health behaviors in Korean adults living alone compared to those living with others. Considering differences in health-related characteristics, people living alone performed less numbers of healthy behaviors in themselves. They made fewer changes related to maintaining a no smoking status or weight control compared to those living with others, but did engage in greater maintenance of alcohol abstinence. Significant factors discovered were “very good” self-rated health status, being overweight, and shorter sleep durations per day, which were all significantly associated with moderate maintenance of multiple healthy behaviors in those living alone.

Our study’s findings showed that people living alone performed fewer healthy behaviors than those who live with others. The percentage of those who engaged in none of the healthy behaviors was significantly higher in people living alone than for those living with others. In particular, previous studies identified only the presence, absence, or degree of health behaviors at the time of the survey [7, 9, 11], whereas this study added further information on the ongoing maintenance of multiple healthy behaviors or attempts to change one’s behaviors as recommended through a comparison of the magnitude in numbers of multiple activities like smoking abstinence, no alcohol consumption, and weight control. However, there were inconsistent associations depending on specific types of maintenance of multiple healthy behaviors. Those living alone made fewer maintenance changes regarding smoking abstinence and weight control compared to those living with others, whereas they showed more maintenance changes regarding no alcohol consumption. Previous studies reported that the smoking rates of adults living alone were generally higher when compared to those living with others [7, 9]. Previous studies have found that smoking is positively associated with cognitive impairment [35] and depressive symptoms [36], which might decrease self–control for maintaining low consumption or giving up smoking. However, some studies reported different results for the alcohol consumption rates of people living alone depending on their age and sex [7, 9]. A study on the changing trends of alcohol consumption rates in Koreans, according to a 2005–2016 panel analysis, uncovered that the daily drinking behaviors of people who live with others were gradually decreasing, while those rates, as well as the frequent drinking rates, of people living alone repeatedly fluctuated and showed more unstable patterns [11]. Therefore, caution is required when comparing the results of prior studies and more evidence related to the numbers of health behaviors in total and specific types of health behaviors on this topic.

Being overweight was a common factor associated with increased numbers of healthy behaviors for both living arrangement groups in this study. Being overweight, for people living alone, demonstrated more significant and positive effects on their maintenance of multiple healthy behaviors, than it did for those living with others. Our findings showed that the rates of being classified as overweight in people living alone were higher when compared to those living with others, and were more than 3% higher than the average overweight rate for Korean adults [24]. A person’s BMI reflects that individual’s eating habits and physical activity, which are both associated with health behaviors [37]. The dietary habits of people living alone are influenced by their health-related lifestyles [22]. In particular, dietary habits including irregular eating times, fast-food intake, frequently eating out, and eating alone increase the likelihood of nutritional imbalances and obesity for people living alone [22]. In general, higher obesity rates increase a country’s socioeconomic burden, and tend to cause a variety of physical and mental health problems for individuals [4]. Therefore, it is carefully customized to develop or implement obesity management plans depending on living arrangements by an individual’s BMI.

In our study, people who live alone reported “very poor” self-rated health statuses at a three times higher rate than those who live with others. Unfortunately, people that live alone tended to engage in more maintenance of multiple healthy behaviors only when their self-rated health status was “very good.” However, people who live with others appear to engage in more maintenance of multiple healthy behaviors— whether they rated their health as good or poor. Consistent with previous studies [38, 39], positive self-rated health statuses of adults living alone were significantly associated with healthier behaviors, such as smoking abstinence, moderate alcohol consumption, adequate sleep durations per day, and positive physical activity. Self-rated health statuses were found to be a factor associated with engagement in healthy behaviors [38], and were also a predictor of both mortality and morbidity, and therefore, can be used to screen high-risk groups [40]. A person’s self-rated health status has been regarded, globally, as providing insight into their general health status and living situation, as it measures mental, psychosocial, and social problems [41]. Therefore, future interventions should consider an individual’s perception and subjective evaluation of their own global health status when developing and implementing health promoting programs targeting people in different living arrangements.

Sleep is a vital component of overall good health [42], that our study confirmed the significant associations with inadequate sleep duration and low maintenance of multiple healthy behaviors. Insufficient sleep quality and quantity have negative associations with health behaviors and their relevant outcomes, as per the results of previous studies [43–45]. Insufficient sleep not only affects behaviors, emotions, and attention but also is associated with risky health behaviors [43, 46]. Interestingly, people who live with others showed a reversed U-shaped association between the maintenance of multiple healthy behaviors and sleep duration per day, as per this study. Previous studies have reported the negative impacts of both too long and too short sleep durations per day on increasing various disease risks [45, 47]. However, only the group demonstrating short sleep durations per day took fewer actions to maintain multiple healthy behaviors when compared to the group reporting adequate sleep duration per day for those living alone in this study. This finding suggests that adequate sleep duration per day is associated with increased numbers of healthy behaviors. Therefore, enhancing a person’s sleep health, in order to develop health promotion strategies in adults, is needed.

The present study confirmed the direction and magnitude of the effects of the factors associated with an individual’s maintenance of multiple healthy behaviors by comparing health behaviors in both the past and present rather than focusing on health behaviors at present or in the past alone. In particular, this study focuses on the group with a vulnerable health status, especially people living alone at risk of engaging in multiple health risk behaviors. People living alone is approximately 15% out of two billion households worldwide [48] and the proportion of people living alone was 30.7% in 2015 according to the Organization for Economic Cooperation and Development [49]. Recently, several countries have developed to assist better health and quality of life for individuals living alone [50–53] as well as South Korea [54]. Korean government provides basic care service (regular safety checks, emotional support, daily life education such as health and nutrition management, or other) and emergency safety care service for the adults living alone [54]. Our study deepens our understanding of how to plan and implement more effective interventions by focusing on modifiable health behaviors. Therefore, health professionals need to consider the characteristics of high-risk health behaviors among people living alone and provide tailored health promotion interventions with modifiable multiple healthy behaviors to prevent chronic illness, increase health benefits, and reduce healthcare costs.

### Study limitations

This study had several limitations. First, KNHANES provides cross-sectional data only and, as a result, our study could not determine causality between the maintenance of multiple healthy behaviors and the associated factors. Second, there is a concern about the reliability of the self-reported information around the smoking, alcohol consumption, and weight control variables. We need to be cautious when handling and defining those health behaviors based on conservative criteria and when using self-reported information, as there are risks of under- or over-reporting as well as recall bias. Third, there is limited information on individuals’ multiple healthy behaviors due to the utilization of secondary data analysis. This study included only data on smoking, alcohol consumption, and weight control, which can compare past and present behaviors in order to define an individual’s maintenance of multiple healthy behaviors or attempts to change behaviors as recommended. We suggest that future studies focus on extensive primary data collection, based on prospective and longitudinal designs considering all age groups of living alone, in defining the key variables for maintaining various healthy behaviors.

## Conclusions

This study examined factors associated with multiple healthy behaviors in Korean adults, based on their living arrangements, using nationally representative data. People living alone performed fewer healthy behaviors when compared to those living with others. Our findings uncovered the differences in the magnitudes of the factors associated with the maintenance of multiple healthy behaviors between people who live alone and those who live with others. Health professionals need to pay attention to the differing natures of modifiable behaviors according to a person’s unique living arrangement. A customized intervention and policy programs according to an individual’s unique living arrangement, as well as multidimensional approach, is needed in order to promote health behaviors in people living alone.

## Supplementary information

**Additional file 1.** Participants’ characteristics related to a single healthy behavior.

## Data Availability

Data for this study were sourced from the KCDC and are available here: https://knhanes.cdc.go.kr/knhanes/eng/index.do

## Abbreviations

aOR: Adjusted odds ratio
BMI: Body mass index
CDC: Centers for Disease Control and Prevention
CI: Confidence interval
IRB: Institutional Review Board
KCDC: Korea Centers for Disease Control and Prevention
KNHANES: Korea National Health and Nutrition Examination Survey
WHO: World Health Organization
