# Supplementary material for: Factors associated with different numbers of health behaviors by living arrangements
Source: BMC Public Health. 2020 Jul 20;20:1141. doi: 10.1186/s12889-020-09242-y (PMC7372790; doi:10.1186/s12889-020-09242-y)
Supplement: Supplementary file 1 — Additional file 1. Participants’ characteristics related to a single healthy behavior. [file 12889_2020_9242_MOESM1_ESM.docx]

| **Additional file 1** Participants’ characteristics related to a single healthy behavior | | | | | | | |
| --- | --- | --- | --- | --- | --- | --- | --- |
| Variables | Categories | **Maintenance of** **health behaviors** | | | | | |
|  |  | No smoking | | No alcohol consumption | | Weight control | |
|  |  | Yes | *P-*value | Yes | *P-*value | Yes | *P-*value |
| Total |  | 76.9%^a,b^  (*n*^c^ =12907) | | 41.6%^a,b^  (*n*^c^ =7494) | | 67.0%^a,b^  (*n*^c^ = 10429) | |
| **Living arrangement** | | | | | | | |
| Living with others | | 77.3 | 0.001 | 41.0 | < 0.001 | 67.6 | < 0.001 |
| Living alone | | 72.0 |  | 48.9 |  | 59.3 |  |
| **Socio-demographic characteristics** | | | | | | | |
| Age | 19–39 | 72.8 | < 0.001 | 32.0 | < 0.001 | 69.5 | < 0.001 |
|  | 40–64 | 76.2 |  | 41.9 |  | 70.6 |  |
|  | 65 and older | 89.3 |  | 65.3 |  | 49.1 |  |
| Sex | Male | 59.0 | < 0.001 | 25.7 | < 0.001 | 64.0 | < 0.001 |
|  | Female | 94.1 |  | 57.0 |  | 69.8 |  |
| Marital status | Single | 71.7 | < 0.001 | 30.4 | < 0.001 | 71.7 | < 0.001 |
|  | Married | 78.4 |  | 44.9 |  | 65.6 |  |
| Education level | Elementary school or lower | 85.8 | < 0.001 | 61.5 | < 0.001 | 52.0 | < 0.001 |
|  | Middle to high school | 73.9 |  | 38.9 |  | 69.7 |  |
|  | College or higher | 77.4 |  | 36.0 |  | 70.9 |  |
| Economic level | Lowest | 78.9 | < 0.01 | 57.9 | < 0.001 | 54.8 | < 0.001 |
|  | Lower-middle, or upper-middle | 75.5 |  | 41.2 |  | 67.3 |  |
|  | Highest | 78.3 |  | 34.2 |  | 72.7 |  |
| Employment status | Unemployed/non-economic activity | 86.5 | < 0.001 | 54.7 | < 0.001 | 66.4 | 0.147 |
|  | Employed | 71.6 |  | 33.8 |  | 67.7 |  |
| **Health-related characteristics** | | | | | | | |
| Self-rated health status | Very poor | 80.5 | < 0.001 | 66.3 | < 0.001 | 55.8 | < 0.001 |
|  | Poor | 73.8 |  | 48.9 |  | 64.2 |  |
|  | Fair | 76.2 |  | 41.7 |  | 66.9 |  |
|  | Good | 80.1 |  | 36.6 |  | 69.7 |  |
|  | Very good | 77.8 |  | 32.1 |  | 70.6 |  |
| BMI | Underweight | 81.2 | < 0.001 | 47.8 | < 0.05 | 54.6 | < 0.001 |
|  | Normal | 78.3 |  | 41.4 |  | 63.8 |  |
|  | Overweight | 73.0 |  | 40.9 |  | 74.8 |  |
| Diagnosis of  chronic diseases | Yes | 81.3 | < 0.001 | 46.4 | < 0.001 | 66.5 | 0.207 |
|  | No | 72.9 |  | 37.0 |  | 67.7 |  |
| Diagnosis of depression | Yes | 78.4 | 0.556 | 51.7 | < 0.001 | 70.8 | 0.071 |
|  | No | 77.1 |  | 41.2 |  | 67.0 |  |
| Stress | Low | 78.8 | < 0.001 | 42.4 | < 0.01 | 67.1 | 0.670 |
|  | High | 71.5 |  | 39.4 |  | 66.7 |  |
| Sleep duration per day | ≤ 5 hours | 76.8 | 0.705 | 47.0 | < 0.001 | 65.0 | < 0.001 |
|  | 6-8 hours | 76.8 |  | 39.8 |  | 68.2 |  |
|  | ≥ 9hours | 78.1 |  | 50.3 |  | 58.4 |  |
| Note: The missing data was less than 5% and non-responses were excluded from the analysis. *P*-values were determined using Chi-square tests with complex sample analysis  *BMI* Body mass index  ^a^Weighted percentages calculated by a complex sample analysis  ^b^These categories were dichotomized using ‘Yes’ and ‘No.’  ^c^Unweighted number | | | | | | | |
